# Quantifying risk factors in medical reports with a context-aware linear model

**DOI:** 10.1093/jamia/ocz004

**Published:** 2019-03-06

**Authors:** Piotr Przybyła, Austin J Brockmeier, Sophia Ananiadou

**Affiliations:** National Centre for Text Mining, School of Computer Science, University of Manchester, Manchester, United Kingdom

**Keywords:** multitask learning, electronic health records, natural language processing, risk assessment, machine learning

## Abstract

**Objective:**

We seek to quantify the mortality risk associated with mentions of medical concepts in textual electronic health records (EHRs). Recognizing mentions of named entities of relevant types (eg, conditions, symptoms, laboratory tests or behaviors) in text is a well-researched task. However, determining the level of risk associated with them is partly dependent on the textual context in which they appear, which may describe severity, temporal aspects, quantity, etc.

**Methods:**

To take into account that a given word appearing in the context of different risk factors (medical concepts) can make different contributions toward risk level, we propose a multitask approach, called context-aware linear modeling, which can be applied using appropriately regularized linear regression. To improve the performance for risk factors unseen in training data (eg, rare diseases), we take into account their distributional similarity to other concepts.

**Results:**

The evaluation is based on a corpus of 531 reports from EHRs with 99 376 risk factors rated manually by experts. While context-aware linear modeling significantly outperforms single-task models, taking into account concept similarity further improves performance, reaching the level of human annotators’ agreements.

**Conclusion:**

Our results show that automatic quantification of risk factors in EHRs can achieve performance comparable to human assessment, and taking into account the multitask structure of the problem and the ability to handle rare concepts is crucial for its accuracy.

## BACKGROUND AND SIGNIFICANCE

The increasing availability of electronic health records (EHRs) enables knowledge discovery^1^ and, with large populations, the ability to identify potential risk factors for key outcomes.^2^ Machine learning techniques, such as deep learning, can leverage comprehensive datasets to predict chronic diseases^3^ or mortality.^4^

Previous analyses have relied on coded EHRs in which each code corresponds to a medical concept (disease, symptom, diagnosis, etc.) linked with a classification system (eg, International Classification of Diseases or Systemized Nomenclature of Medicine–Clinical Terms). Many EHRs, however, include additional text, which, although requiring further processing, is more subtle and fine grained than a coded concept. Moreover, research has shown that the free-text information may be more reliable than codes.^5^^,^^6^ Therefore supplementing code-based classifiers with information extracted from free-text EHRs may improve outcome predictions.^7^^,^^8^

Extracting information relevant for mortality risk assessment from textual EHRs requires addressing 2 main challenges. First, to recognize mentions of medical concepts, such as conditions, symptoms, or treatments that could be considered risk factors—each associated with a different baseline mortality rate (high for *heart attack*, low for *common cold*). Second, to assess how risk is influenced by the textual context, which may describe severity (*mild*, *critical*), temporal aspects (*recently*, *5 years*), quantity (*120/80*, *36.6*), other patients (*family history*, *brother*), anatomy (*toe*, *cranium*), etc. Clearly the occurrences of contextual words are crucial when assessing the severity of a risk factor. While some words (*mild*, *severe*, *abnormal*) influence risk estimate for all concepts similarly, other words have a context-dependent interpretation (*decreased* can be a cause for concern in the case of hemoglobin, but not for low-density lipoprotein cholesterol). Therefore, risk-prediction models would benefit from the use of both context-dependent and context-independent features.

Our goal is to predict the mortality risk level associated with medical concept mentions in free-text EHRs. We approach this as a machine learning problem, which involves a number of challenges, including feature sparsity and high-dimensionality: there are tens of thousands of possible word-based features, only a few of which are present in any given instance. Additionally, the task space is sparse: there are thousands of possible risk factors, and training examples will not cover all cases. This implies that when applied to new examples, the model would have to estimate the level of risk for previously unseen concepts.

To address these issues, we approach risk-level estimation as a multitask learning problem, in which each task corresponds to a different risk factor. We introduce context-aware linear modeling (CALM), a multitask learning framework that balances learning the contributions of task-dependent and task-independent features. In this framework, transfer learning applied to unseen tasks relies on both the task-independent features and a dense vector representation of tasks that approximates the distributional similarity between medical concepts. We show how the inference problem can be cast as L_1_-norm regularized linear regression (LASSO),^9^ enabling efficient estimation for high-dimensional and sparse data. We evaluate this approach using a collection of 99 376 mentions of 9988 risk factors manually annotated with the level of risk and obtain performance comparable to the level of agreement between human experts.

### Risk factor extraction from textual EHRs

The problems of extracting information from free-text clinical documents have been thoroughly investigated,^10^ giving rise to a plethora of tools^11^ and other resources.^12^ While recognizing and normalizing medical concept mentions are relatively well understood,^13^^,^^14^ taking into account information in the neighborhood of a mention requires more sophisticated processing such as temporal analysis,^15^ negation recognition,^16^ assertion classification (eg, present, missing, other patient),^15^ uncertainty estimation,^17^ and parsing numerical results.^18^ Current solutions to these problems often rely on manually created heuristics.

Specifically with regard to risk assessment, taking into account the textual context of a mention (other than negation) is relatively rare. Expressions such as “risk increase” or “60% risk” can be used for extracting factors affecting the likelihood of a disease from healthcare guidelines^19^ or medical literature,^20^^,^^21^ whereas understanding temporal aspects and linking laboratory tests with numerical results are necessary to recognize mentions of prespecified risk factors for cardiac artery disease (2014 i2b2 shared task)^22^ or symptoms of appendicitis.^23^

Our approach differs from these works by focusing on mortality in general instead of a specific disease. This means that there are numerous potentially relevant concepts instead of a fixed list of risk factors. Moreover, it is infeasible to manually prepare regular expressions to assess the risk level for every factor. Thus, contrary to previous approaches, our solution is driven purely by machine learning and exploits aspects of the problem’s multitask structure.

### Multitask learning

Multitask learning is a paradigm of machine learning aimed at situations in which there are several learning tasks, which are presumed to be related, and could therefore benefit from joint learning.^24–26^

In this study, an individual learning task corresponds to estimating the mortality risk for all mentions of a unique medical concept. While the set of tasks shares the same feature set and output values, the number of instances for each task differs as does the baseline risk and the effect of different features. For example, in the context of a laboratory test, the occurrence of the word *abnormal* would increase the associated risk, while the effect of specific numbers (or looser quantitative categorization like *high* or *low*) would depend on the specific test. Ideally, the model should contain both features that consistently affect the risk across all tasks, and task-specific features when there is sufficient evidence to justify them.

Multitask learning covers many distinct machine learning scenarios.^27^ However, in all cases, the potential benefit relies on the degree of relatedness between tasks. Learning multiple tasks in parallel is useful only if the models for different tasks can be compactly represented together. One can then constrain the complexity of the models such that either novel tasks^28^ or a fixed set of tasks can be efficiently learned.

In this regard, neural networks form internal representations from features that are shared across tasks.^24^^,^^25^ Local sharing of the factorized weights for every neuron can also be beneficial.^29^ More flexible information sharing between tasks can be exploited using empirical Bayes methods^30–35^ for neural networks or linear models.

Linear models are well studied in the context of multitask learning. They remain competitive to more complex models for high-dimensional sparse data with limited training instances and allow problem-specific model assumptions and regularization schemes.^36^ For instance, the coefficient vectors for different tasks can be regularized by penalizing their distance to a common coefficient vector.^36^ The difference between any pair of tasks can also be constrained.^37^ Our linear model is similar but distinct, as we measure closeness to a common vector using the L_1_ norm, while also penalizing the L_1_ norm of the common vector.

Rather than restricting the coefficient vectors individually, many studies have used the L_2,1_ norm applied to the matrix of coefficient vectors to encourage common subset selection across tasks (reducing the number of unique features for task-specific features).^38–40^ This regularization is a structured case of group LASSO,^41^ in which a group corresponds to a feature across all tasks. However, this regularization neither encourages the similarity of coefficients between tasks nor limits the number tasks using a selected feature, thus reducing the model interpretability.

Alternatively, task-specific coefficient vectors can be approximated as linear combinations of a small set of shared vectors.^25^^,^^42^^,^^43^ A similar effect with better optimization guarantees can be achieved by using trace-norm regularization on the matrix of coefficient vectors.^44–47^ The optimization for the L_2,1_-norm and trace-norm regularizations are related,^39^^,^^40^ but the latter does not perform feature selection.

To deal with multiple assumptions regarding the varied relationships between features and outputs across the tasks, multiple constraints are needed in parallel.^48^ Similar to our model, dirty models^49^ assume the coefficients are a combination of 2 sets: one with L_2,1_-norm regularization and another with L_1_-norm regularization for task-specific features. Different assumptions may need other combinations of regularizations.^50^

## MATERIALS AND METHODS

### Risk factor analysis

This study is part of an effort to automatically assess the mortality risk of an individual based on textual health records. Toward this goal we aim to recognize and determine the level of each individual risk factor occurring within a given text. By *risk factor* we mean any medical concept mention which, when encountered in a patient’s medical records, should be taken into account to predict their lifespan. Therefore, we include both mentions that influence mortality directly (eg, *heart attack*, *diabetes*) and more indirect clues of a risk (eg, *cardiologist*, *MRI*).

After preliminary analysis and discussion with 4 domain experts, we decided to assess the following categories of the most common and significant risk factors:

- Symptom: any simple abnormality in bodily state or function that could be observed by a patient
- Laboratory test: a procedure analyzing patient’s bodily fluids or tissue
- Condition: diseases, injuries, and other abnormalities detectable through medical investigation
- Investigation: any procedure performed to investigate patient’s condition (other than a laboratory test)
- Behavior: a patient’s habits that may affect their health
- Healthcare provider: an institution, organizational unit or a medic providing medical service
- Treatment: a procedure intended to improve a patient’s state.

Each risk factor mention is assigned a qualitative measure of the risk severity given the context, which is limited to the current sentence. The risk level is encoded as 1 of 4 possible categories:

- None: the factor does not influence the patient (eg, it is negated or associated with unrelated person)
- Low: the factor applies to the patient, but does not pose a significant mortality risk
- Medium: the factor is associated with mild or moderate mortality risk
- High: the factor is associated with major mortality risk

### Analysis workflow

**Figure 1.**
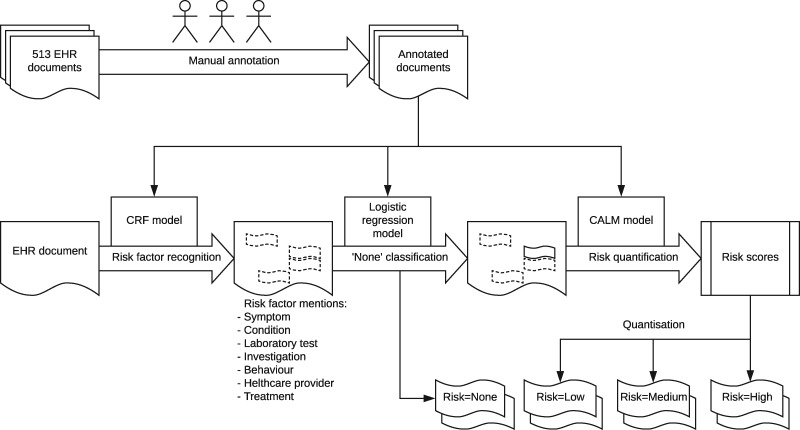
Outline of the risk assessment workflow. The focus of this article is on the last stage (ie, risk quantification). CALM: context-aware linear modeling; CRF: Conditional Random Field; EHR: electronic health record.

Our risk extraction workflow, shown in Figure 1, consists of the following steps:

1. annotating a corpus of medical records with the desired categories using human experts
2. training a named entity recognizer to extract factors of the given categories
3. training a binary classification model that filters out factors with risk level none.
4. training a model that estimates the risk level for non-none risk factor mentions (the focus of the proposed methodology).

The annotation process was carried out in close cooperation with experts in medical risk assessment. First, annotation guidelines were developed through discussion with the experts and trial annotations to ensure the rules are straightforward and cover the important risk. Second, a corpus consisting of anonymized health records was gathered. The majority of content (300 reports, 80% of risk factors) was obtained by extracting discharge summaries from the openly available MIMIC-III database, which had been de-identified and approved for release.^51^ This dataset, produced by critical care units, frequently describes very serious medical problems. To balance it with some lower-risk content, it was extended with a synthetic set of 231 documents (20% of risk factors) by asking the experts to generate free-text health reports in the style produced by general practitioners for routine medical history summarization.

All the manual annotation was performed within *brat.*^52^ To expedite the process, the corpus was preannotated by mapping concepts recognized by UMLS MetaMap^53^ to risk factor categories. Three annotators took part in the annotation. After an initial annotation round, documents were selected for annotation by applying active learning with a named entity recognition model. This means that in each subsequent round a named entity recognition model was trained on annotations available then and applied to the unlabeled documents. Next, a model’s uncertainty about each document was computed by averaging the entropy of label probabilities across tokens. The documents with the highest average entropy (the lowest model certainty) were chosen for the next round of manual annotation. In total, of 531 annotated reports, 121 were double-annotated (approximately one-third by each annotator pair) to assess interannotator agreement between them. Interannotator agreement for entity extraction, measured using the relaxed F-score (considering 2 entities matching if their spans overlap), reached a value of 0.865. To measure risk assignment agreement, we computed accuracy on the matching entities and obtained a value of 77%. For the purpose of risk quantification evaluation baseline (see Results), the risk levels were converted to continuous values as described in the next section and used to compute mean squared error.

The main focus of this article is on the last stage of the workflow, namely risk quantification. The Supplementary Appendix provides information on the previous stages and evaluation of the performance of the entire workflow.

### Risk quantification

The goal of risk quantification is to select a risk level (low, medium, or high) for each non-None risk factor mention in context. Due to their intrinsic ordering, we represent them quantitatively (low as 0.0, medium as 1.0, high as 2.0) and consider their prediction as a regression task. The trained model will output a real-valued score, which is mapped to low, medium, or high by choosing appropriate threshold values.

The risk quantification problem is informed by 2 sources: the risk factor concept itself and neighboring words. When MetaMap Lite^54^ recognizes a concept overlapping with the mention, the corresponding UMLS concept ID is used; otherwise, a surrogate ID is formed by concatenating the included words (ignoring capitalization). A concept is represented in the model using one or both of the following:

- a sparse representation in which every concept ID is represented with a separate binary feature,
- a dense vector from continuous (L_2_-normalized) UMLS concept embeddings trained on a large corpus^55^

The latter allows us to improve the performance in the case of concepts unseen in training data, by leveraging their distributional similarity to known concepts.

Each mention in a sentence is represented using the following features:

- the category label for the mention,
- the bag-of-words representation of lemmata (base forms) of up to 5 words preceding the mention,
- the bag-of-words representation of lemmata of up to 5 words following the mention,
- the value of the logarithm of the first number following the mention (within 4 words), discretized by dividing the range between –5.0 and 10.0 (manually selected to cover the vast majority of encountered numbers) into 50 equal-sized sections.
- mention attributes (negation: affirmed or negated, experiencer: patient or other, and temporality: historical, recent, not particular) recognized by ConText,^56^ a tool based on regular expressions.

All features are represented with binary indicator variables.

### Context-aware linear modeling

Examples in the training set are triplets (t,x,y)∈𝒯×𝒳×R consisting of the task identifier, the features, and the target value, where 𝒯={1,…,T} is the set of tasks (corresponding to concept IDs), $\text{X}=R^{d}$ is the feature space, and the target values are real-valued risk scores. The tasks also index the task embeddings $\text{ℰ}={\left\{e_{t}\right\}}_{t\in \text{𝒯}}$, where tasks lacking a trained embedding vector are mapped to a vector of zeros.

For a previously unseen task, with unknown task embedding, the model prediction of the risk is computed as

(1) $$\hat{y}=b+u^{\text{⊤}}x,$$

where b∈R is a bias, $u,x\in R^{d}$ are the common coefficient vector and feature representation. For tasks unseen in the training set, but with known embedding, the prediction is

(2) $$\hat{y}=b+u^{\text{⊤}}x+v^{\text{⊤}}e_{t},$$

where $v,e_{t}\in R^{d_{e}}$ are the task-embedding coefficients and task embedding. Since the task embedding is constant for every instance of a task, $v^{\text{⊤}}e_{t}$ acts like a bias for the task. The full context-aware model prediction is expressed as

(3) $$\hat{y}=\underset{\text{shared}}{b+u^{\text{⊤}}x+v^{\text{⊤}}e_{t}︸}+\underset{\text{task-specific}}{b_{t}+w_{t}^{\text{⊤}}x︸},$$

where $b_{t}\in R$ is the task-specific bias and $w_{t}$ is the task-specific coefficient vector.

The vector of task-specific biases and matrix of task-specific coefficients in the training set are denoted $b_{\text{T}}={\left[b_{1},b_{2},\ldots ,b_{𝒯}\right]}^{\text{⊤}}$ and $W=[w_{1},w_{2},\ldots ,w_{T}]$, respectively. Equation 3 is linear with respect to all of the parameters $b,u,b_{𝒯},v,W$. The total number of parameters is $1+d+d_{e}+T\cdot (1+d)$. The parameters are optimized to minimize the mean squared error of the risk estimate in the training set. This corresponds to a maximum likelihood estimate assuming the predictions errors ε have a zero-mean Gaussian distribution with some variance $\sigma^{2}$, such that the risk is expressed as $\hat{y}+\text{ε}$.

For an intelligible model, the goal is to select a subset of features that are common (shared) across tasks at the same time as selecting a subset of features that have a task-specific relationship. The subset of task-specific features could be common across tasks,^38–40^ or each task could have an independent subset.^49^

These diverse constraints can be enforced during training using regularization terms on the parameters. Collecting the parameters into the vector $u\prime ={\left[u^{\text{⊤}},v^{\text{⊤}}\right]}^{\text{⊤}}$ and the matrix $W\prime ={\left[b_{\text{𝒯}},W^{\text{⊤}}\right]}^{\text{⊤}}$, we propose to balance the regularization terms and the mean squared error loss using trade-off parameters ψ and λ:

(4) $$\underset{b,u,v,b_{\text{𝒯}},W}{\text{min}}\sum_{i=1}^{n}\frac{1}{n}{\left(y_{i}-{\hat{y}}_{i}\right)}^{2}+\frac{\lambda }{1-\psi }\Omega_{1}(u\prime )+\frac{\lambda }{\psi }\Omega_{2}(W\prime ).$$

This formulation is valid for values of ψ between 0 and 1. When ψ=0 it reduces to single-task learning (an infinite penalty on the $\Omega_{2}$ term forces the task-specific coefficients to be zero), and when ψ=1 this is a multitask model without any shared coefficients.

To select a subset of features, the common coefficients and the task-embedding coefficients are regularized with the sparsity-inducing L_1_ norm ($||\cdot |{|}_{1}$):

(5) $$\Omega_{1}(u\prime )=\|u{\|}_{1}+\varphi \|v{\|}_{1},\;\Omega_{2}(W\prime )=\|\text{vec}(W\prime ){\|}_{1}=\sum_{i=1}^{d}\sum_{j=1}^{T}\left|W\prime_{ij}\right|,$$

where φ is set to 1 enable the task embedding, or set to infinity to remove the task embedding from the model.

The task-specific biases and coefficients can be regularized with either the L_1_ norm or combinations of structured matrix norms. For instance, the L_2, 1_ norm will encourage the selection of features that are relevant across all tasks (ie, the task-specific coefficients for a given feature are either zero for all tasks or nonzero for all tasks). Thus, this regularization scheme is equivalent to a group LASSO where the task-specific coefficients for a single feature form a group:

(6) $$\Omega_{2}(W\prime )=\|W\prime {\|}_{2,1}=\sum_{i=1}^{d}\sqrt{\sum_{j=1}^{T}{\left(W_{ij}^{\prime }\right)}^{2}}.$$

Both of these regularization schemes lead to convex minimization problems. In particular, using the L_1_ norm for each regularization term,

(7) $$\frac{\lambda }{1-\psi }\left(\|u{\|}_{1}+\varphi \|v{\|}_{1}\right)+\frac{\lambda }{\psi }\left(\sum_{t}\left|b_{t}\right|+\|w_{t}{\|}_{1}\right),$$

encourages only a subset of the common features and the task specific features and biases to be nonzero. Due to the high-dimensional sparse data, we use glmnet^57^ to solve the model with the L_1_ + L_1_ regularization. The values of ψ and λ are chosen via 5-fold cross validation on the training set. For a given value of ψ∈{0,0.05,…,0.95,1} the coefficients across a range of λ values are computed efficiently via the regularization path.

Alternatively, the structure of the optimization problems for L_2,1_ or trace-norm regularizations requires special solvers for large-scale problems^46^^,^^58^^,^^59^ or general optimization frameworks such as alternating direction method of multipliers approaches.^60–64^ In particular, we implement an alternating direction method of multipliers approach to estimate the model coefficients for a regularization expression combining the L_1_ norm on the common factors and the L_2,1_ norm for multitask feature selection:

(8) $$\frac{\lambda }{1-\psi }\left(\|u{\|}_{1}+\varphi \|v{\|}_{1}\right)+\frac{\lambda }{\psi }\|W\prime {\|}_{2,1},$$

where $\frac{\lambda }{1-\psi },\frac{\lambda }{\psi }\in {\left\{10^{i}\right\}}_{i=-2}^{2}$ are chosen via 5-fold cross-validation.

### Evaluation

**Figure 2.**
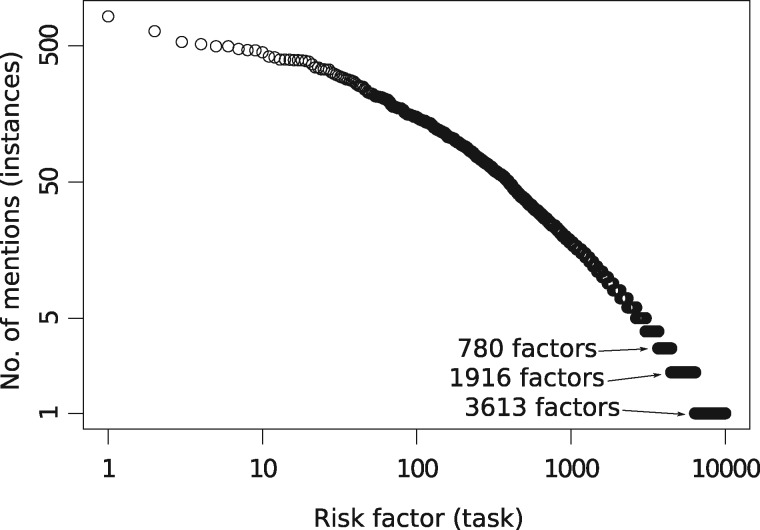
Risk factor mention count distribution plotted on log-log scale.

To evaluate the CALM method on the risk quantification problem, we use the corpus described in the Analysis Workflow section, which contains 99 376 risk factor mentions assigned a risk level other than None. In total, 21 776 binary features are generated; there are 9988 distinct risk factors (tasks). Figure 2 shows the uneven distribution of the number of instances among the tasks following Zipf’s law,^65^ typical for word frequencies: from a few common risk factors (the most frequent has 824 mentions) to 3613 single-mention ones. 6534 of the risk factors are assigned a UMLS ID and 5114 of these have concept embeddings.

To allow us to focus on the problem of risk quantification, the risk factors and their risk levels are taken from the human annotations (evaluation of an end-to-end solution, where risk quantification is run on automatically recognized mentions, is provided in the appendix). The data is divided into a training (70%), development (15%) and test set (15%, 79 documents, 11 596 mentions) for the final evaluation. The division is random, and stratified, such that the ratio of MIMIC summaries and manually created reports is consistent in each portion and all double-annotated documents are in the training set. Due to the distribution of risk factors, 8.88% of the test instances correspond to risk factors that only appear in the test data.

The evaluation measure is the mean squared error (MSE) $\frac{1}{n}\sum_{i}{\left(y_{i}-{\hat{y}}_{i}\right)}^{2}$. Two baselines are available:

- Naive: MSE between the test instances and the mean risk in the training data
- Human: MSE between pairs of risk factor mentions in double-annotated documents

The naive baseline represents the performance of a simplistic model that assigns the same risk value (mean risk from the training portion) to every single instance in the test data and the human baseline indicates the performance that we could expect from a human (measured through interannotator agreement).

To assess the significance of differences in MSE between compared methods, a randomized permutation test^66^ was performed. Specifically, to compare outputs of methods A and B, a set of R surrogate pairs A’ and B’ was created by swapping the predictions of A and B for randomly selected documents. Next, let r denote the number of surrogates for which the absolute difference of the performance measure (ie, MSE) was at least as large as the original difference between A and B. For large R (we used 100 000), the value computed as $\frac{r+1}{R+1}$ approaches the significance level and we treat .01 as the threshold for significance.

## RESULTS

Table 1 shows the MSE for different approaches, comparing baselines (human agreement and mean risk in the training data), single-task models and multitask CALM. The table includes the MSE computed on subsets of risk factors present in the training data (known) and only in testing (unknown). The results show that taking into account the problem’s multitask structure reduces the error substantially: MSE falls from 0.33 to 0.25. This is mostly due to better performance on known tasks, as the error on unknown ones is lower with the single-task approach (0.40) compared with CALM (0.45). The addition of the task embeddings reduces this gap, achieving the lowest overall error of 0.2425, which is better than the human agreement level over double-annotated documents. The value of ψ selected through 5-fold cross-validation for this configuration is 0.5, which corresponds to an equal penalty for the common and task-specific feature coefficients. The randomized permutation test indicates statistical significant differences (significance level of .01) between the overall MSE of the best method (L_1_ + L_1 _with embeddings) and each of the other methods (*P* < 10^−5^), except for multitask feature selection.

Due to the high number of both tasks and features, the total number of coefficients is over 182 000 000. Table 2 shows the number of nonzero coefficients in the 3 models that use embeddings. We can see that the 2 best-performing models differ greatly, mostly due to number of task-specific coefficients: L_1_ + L_1 _is a simpler model, making it more usable and interpretable.

To provide greater insight into the best-performing model, Table 3 lists the most important features based on the magnitude of the feature coefficient. For example, many of the common features with negative coefficients are words related to family (*fh*, *father*), since a condition mentioned in the context of family history carries less risk than if it was experienced by the patient. In contrast, the appearance of the word *critical* increases the risk of the following mention. The task-specific features frequently correspond to laboratory tests, with the risk depending on the following numerical expression. They can also describe more subtle phenomena (eg, the word *due* increases risk associated with *alcohol*, as an expression such as “likely due to alcohol abuse” suggests that alcohol problem is serious enough to cause other factors).

**Table 1.** Error values for baseline approaches and different CALM methods (with their regularization schemes) calculated on all mentions in the test set (all) and the subsets of mentions with risk factors present or absent in the training data (known and unknown, respectively)

			MSE	
Method	Regularization Scheme	All	Known	Unknown
Human baseline (interannotator agreement)	–	0.2465	—	—
Naive baseline (mean risk in training data)	–	0.5362	0.5335	0.5631
Single task without embeddings	Eq. 7, φ=∞, ψ=0, λ=0.002636	0.3295	0.3228	0.3978
Single task with embeddings	Eq. 7, φ=1, ψ=0, λ=0.002432	0.2977	0.2908	0.3680^a^
L_1_ + L_1_ without embeddings	Eq. 7, φ=∞, ψ=0.55, λ=0.002116	0.2533	0.2345	0.4454
L_1_ + L_1 _with embeddings	Eq. 7, φ=1, ψ=0.5, λ=0.002432	0.2425^a^	0.2289^a^	0.3817
Multitask feature selection	Eq. 8, φ=1, $\psi =\frac{1}{11}$, $\lambda =\frac{10}{11}$	0.2461	0.2306	0.4052

CALM: context-aware linear modeling; MSE: mean squared error.

^a^lowest error values in each set.

**Table 2.** Number of available coefficients in each of the elements of CALM models and number of nonzero values in 3 of the methods employed

	**Nonzero Coefficients**				
	u Common Feature Coefficients	$b_{𝒯}$ Task-Specific Biases	v Task Embedding Coefficients	*W* Task-Specific Coefficients	**All**
Available	19 835	9215	500	182 779 525	182 809 075
Single task with embeddings	2118	—	94	—	2212
L_1_ + L_1 _with embeddings	1928	2000	77	7735	11 740
Multitask feature selection	2656	9215	37	63 319	75 227

CALM: context-aware linear modeling.

**Table 3.** Top 11 features with the highest importance for the model (features with at least 5 occurrences selected according to absolute coefficient value) of 2 types: common and specific to a risk factor (quoted as appearing in text)

Risk Factor	Feature	Coefficient	Explanation
(common)	<lumbosacral	–0.7471	Abnormalities in the lower back have a low mortality risk
(common)	>her2	+0.6864	Overexpression of HER2 associated with breast cancer
(common)	<fh	–0.6606	A risk factor listed as family history (fh) carries lower risk
(common)	<pint	+0.6443	Alcohol quantity influencing the risk
(common)	<lid	–0.6131	Treatments or symptoms near an eyelid have a lower mortality risk
(common)	<critical	+0.6077	Critical state of a condition
(common)	<vodka	+0.6047	Indicating higher alcohol consumption
(common)	<subdiaphragmatic	–0.5761	Increased risk for factors regarding the body cavity below the diaphragm
(common)	>detox	+0.5620	Mentioned in context of alcohol or drug problems
(common)	<methadone	+0.5549	An analgesic that can be administered to treat withdrawal from illicit drugs
(common)	<father	–0.5368	Conditions experienced by the patient’s father
BUN	[40–55]	+0.9039	A high level of blood urea nitrogen (mg/dL)
CAD	ConText.OTHER	–0.7943	Risk of coronary artery disease lessened when experienced by someone other than the patient, such as family member
Alcohol	<due	+0.7823	Alcohol abuse causing other condition, as in *due to alcohol use*
BUN	[55–74]	+0.7657	A high level of blood urea nitrogen (mg/dL)
EtOH	[445–601]	+0.7510	Alcohol level measured on admission (mg/dL)
Smoking	>day	+0.7380	Smoking daily (eg, *smoking 23/day*)
BP	[181–245]	+0.7375	Increased risk from high systolic blood pressure (mm Hg)
Varices	>leg	–0.7134	Varices, when located in legs, carry less risk
SBP	[181–245]	+0.7120	Increased risk from high systolic blood pressure (mm Hg)
Triglycerides	[2.72–3.67]	+0.7119	Increased triglyceride level (mmol/L)
Bnzodzp	>pos	+0.7043	Positive results of benzodiazepines measured on admission, possible drug abuse

Features include several types: numbers falling in specific ranges (square brackets), words in left or right neighborhood (<, >) and labels assigned by the ConText tool.^56^

BP: blood pressure; BUN: blood urea nitrogen; CAD: coronary artery disease; EtOH: ethanol; SBP: systolic blood pressure.

**Figure 3.**
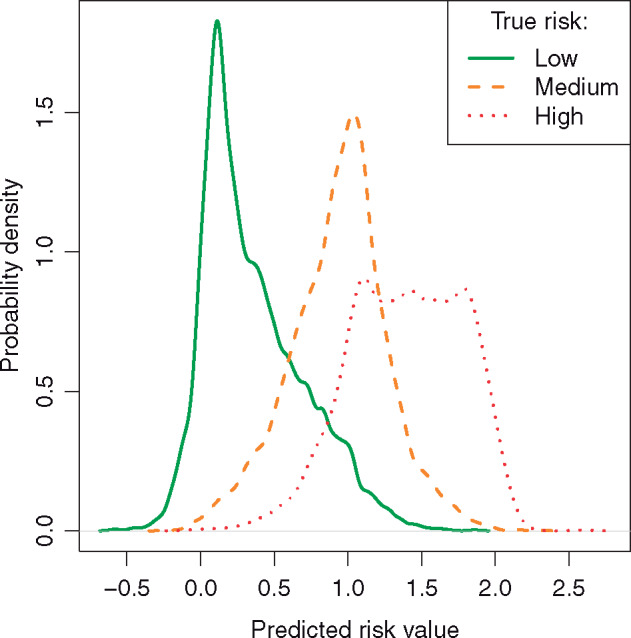
Empirical probability density function of the risk values predicted by the best-performing model for the risk factors belonging to the low, medium, and high category according to manual annotation.

To provide more insight into the type of errors made by the best-performing model, Figure 3 shows the empirical probability density plot of the predicted risk values for the mentions annotated as low, medium, or high by the experts. We can see that the higher true risk level is, the harder it is for the model to assess it properly. While low-risk factors are well separated from the rest, distinction between medium and high remains a bigger challenge, especially given the underestimation and the range of predictions associated with high-risk factors.

To illustrate the results in terms of discrete categories of low, medium, and high risk, we performed quantization of the continuous risk score using the manually selected thresholds:

Low < 0.5 ≤ Medium < 1.3 ≤ High.

To compare the differences between predicted and actual risk levels with differences between annotators we computed confusion matrices, which are shown in Table 4. They show that the CALM model achieves better performance for medium-level risk factors and is less likely to assign low or high values compared with human annotators. This could be linked with the loss function associated with the linear regression in CALM imposing a lower penalty for confusing low or high with medium than with the alternative.

**Table 4.** Confusion matrices showing the disagreements in the test set (between the best CALM model’s predictions and manual annotation) and in the double-annotated part of the corpus (aggregated across all pairs of 3 annotators)

		Actual (%)					Annotator B (%)		
		Low	Medium	High			Low	Medium	High
Predicted	Low	70.29	**9.86** **^a^**	**1.24** **^a^**	Annotator A	Low	**83.19** **^a^**	11.88	1.33
	Medium	28.92	**78.41** **^a^**	39.74		Medium	**15.93** **^a^**	75.02	**26.57** **^a^**
	High	**0.80** **^a^**	**11.72** **^a^**	59.03		High	0.88	13.09	**72.10** **^a^**

CALM: context-aware linear modeling.

^a^better value (higher agreement or lower disagreement).

**Figure 4.**
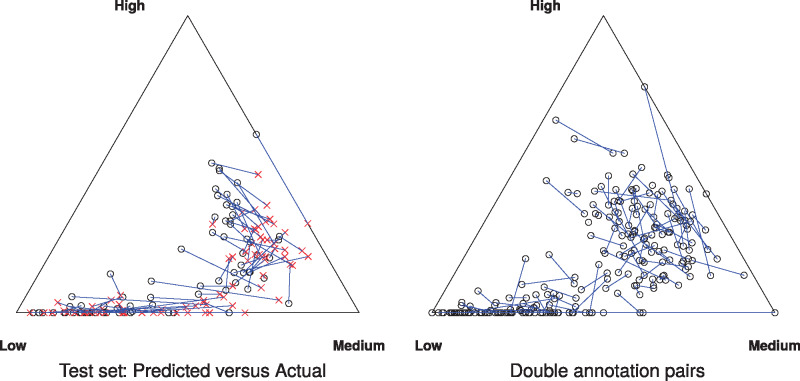
Proportion of low-, medium-, and high-risk factors in electronic health record documents (the corners correspond to documents with risk factors all marked at the same risk level.) For the test set, the actual values (black circles) are compared with the context-aware linear modeling predictions (red crosses). Pairs of human annotators are compared on a subset of the training set.

Finally, Figure 4 shows the differences in terms of the proportion of high-, medium-, and low-risk factors in each document displayed as ternary plots. Consistently with the confusion tables, we can see the systematic bias of the model to assign the medium risk value, compared with the variety of directions caused by interannotator disagreements.

## DISCUSSION

The results demonstrate that the multitask nature of the problem can be successfully captured by the CALM framework. CALM performs better than a single-task model, and is able to generalize to new tasks by exploiting the similarity of the risk assessment for similar risk factors. Two of the regularization schemes, namely the L_1_ + L_1 _scheme and the multitask feature selection, achieve similar error values, but differ in terms of model complexity. Given that the L_1_ + L_1_ model uses less than one-sixth of the coefficients, it would be preferable in scenarios where interpretability of model decisions is a factor.

Any supervised model is limited in its accuracy by the consistency of the training data. For this data set, the model achieved an error rate on par with the level of disagreement between human experts. However, specific discrepancies in the model’s risk assessment remain, as shown by the aggregated summaries of the risk categories (Table 4) or documents (Figure 4). For example, 40% of the high-level risk factors were assigned the medium level by the model. As in most applications, the cost of overestimating a risk is lower than underestimating it, the MSE cost function could be replaced by an asymmetric cost function that penalizes underestimation of risk. Alternatively, the risk levels may be better modeled with ordinal regression, with appropriate regularization.^67^ The interannotator discrepancies shown in Table 4 and Figure 4 show that the human experts also had difficulties in agreeing on risk levels. The risk scale we used is relatively coarse-grained and some risk factors that are currently annotated as high risk may in fact differ in significance in human judgement, so adding finer-grained risk levels (e.g., medium-high and very high) or quantitative judgements of expected impact to lifespan may be beneficial.

Based on this outcome, future work should incorporate the feedback of the experts and use their knowledge to perform detailed manual error analysis. This would help to understand, which of the prediction errors are caused by imperfections of the algorithm and which are caused by the differences of subjective risk judgement. Enhancing the quality and consistency of annotations using limited resources could be facilitated by using annotator reliability assessment methods, such as proactive learning,^68^ and supporting annotation tools.^69^ Improving the performance of other elements of the end-to-end solution could also result in better risk assessment (see the Supplementary Appendix for details).

In a broader context, the current model is limited to assessing individual statements because it only considers the features within the same sentence. Assessing individual statements is useful to pinpoint risks, but to move toward overall mortality risk the context of the whole medical record across multiple visits could be considered. In this case, the subjectivity of human judgement could be avoided by using patient survival data instead. However, a classifier using an entire medical history, which is itself extremely difficult to obtain, may become biased toward serious conditions occurring late in the record and ignore earlier and less serious risk indicators. Using expert judgement allows us to identify known risk factors within portions of reports, even if their effect is subtle. To move beyond individual statements, the association between different risk factors per record could be captured by introducing aspects of collaborative filtering,^70^ another direction for future work.

## CONCLUSION

In this work, we have introduced a novel framework, CALM, and used it to categorize individual mentions of risk factors in free-text EHRs as having a high, medium, or low level of mortality risk, depending on both the medical concept to which they refer and the lexical context in which the mentions occur. To assess the influence of both features that are independent of risk factors and those which are specific to individual risk factors, we cast risk quantification as a multitask learning problem, which benefits from prior information in the form of concept embeddings. Experimental results validate our approach: the performance of our multitask approach is better than the single-task approach and approaches the level of interannotator agreement.

## FUNDING

This work was supported by the Manchester Molecular Pathology Innovation Centre (MRC grant number MR/N00583X/1) and by Pacific Life Re.

## AUTHOR CONTRIBUTORS

PP and SA contributed to the conception and design of the project. PP coordinated the annotation process and performed the feature generation. The machine learning models were designed, implemented and evaluated by PP and AJB. PP and AJB drafted the manuscript, and all authors reviewed, provided input, and accepted the submitted version.

*Conflict of interest statement*. None declared.

## Supplementary Material

Supplementary_Appendix_ocz004
