# Supplementary material for: Quantifying risk factors in medical reports with a context-aware linear model
Source: J Am Med Inform Assoc. 2019 Mar 6;26(6):537–46. doi: 10.1093/jamia/ocz004 (PMC6515525; doi:10.1093/jamia/ocz004)
Supplement: Supplementary_Appendix_ocz004 [file supplementary_appendix_ocz004.pdf]

Quantifying Risk Factors in Medical Reports with a Context-Aware Linear Model Appendix: End-to-End Solution and Evaluation

Piotr Przybyła, Austin J. Brockmeier, Sophia Ananiadou
National Centre for Text Mining, University of Manchester, UK
sophia.ananiadou@manchester.ac.uk

1 Introduction

This appendix describes a complete automated approach for risk assessment. It is an *end-to-end* process, since it takes free-text electronic health records as an input and outputs a list of encountered risk factors with their risk values. The description of the complete process provides context to the risk quantification task, i.e. assigning a score to each of the risk factors found in a medical report, which was the focus of the main body of the article.

Risk assessment is accomplished through a sequence of three stages (see Figure 1 in the main body of the article for illustration):

1. Risk factor recognition,
2. Risk classification,
3. Risk quantification,

Stage 3 is thoroughly discussed in the main body of the article; here information on stage 1 and 2 are provided with evaluation results of the whole process.

2 Methods

2.1 Risk factor recognition

Risk factor recognition was approached as a standard Named Entity Recognition (NER) task, where the purpose is to find all the mentions of words and multiword expressions that fall into predefined categories using manually annotated training data. The corpus (see the main body of the article for more information on the annotation process) contains 531 documents with 109,856 entities, which were divided into train and test subsets.

In the first preprocessing step, the documents were split into sentences using *GENIA* sentence splitter [10], tokenised using *OSCAR4* [5] and POS-tagged using *GENIA* tagger[13]. Secondly, *MetaMap Lite* tagger [2] was used to recognise UMLS concepts belonging to semantic types corresponding to risk factor categories. Thirdly, word clustering, demonstrated to improve NER performance [1], was prepared: specifically, word embedding vectors were computed by applying *word2vec* [7] to all the MIMIC discharge summaries (unused in the annotated corpus) and clustered using k-means [4] implemented in *ClusterR* package [11] in *R* [9]. Eight different clusterings were generated by computing word vectors using a context window of 2 or 5 tokens and running k-means with the number clusters equal 128, 256, 512 or 1024. Finally, regular expressions were used to mark lines that have formatting typical for headers.

As a result, the following features were available for each token:

- surface form text,
- word lemma,
- POS tag,
- BIO label of the recognised UMLS concepts with information about semantic type, e.g. `B-dsyn`,
- label denoting if the token belongs to a line recognised as a header,
- cluster identifiers in each of the eight clusterings,

The features based on lemmas and UMLS tags were taken into account through unigrams and bigrams in the close neighbourhood of the target token.

These features were then used to build a Conditional Random Fields (CRF) [6] model of BIO-encoded mentions of risk factors (with categories). For this purpose the *CRF++*[1] software was used with L1-norm regularisation.

2.2 Risk classification

The purpose of risk classification is to classify a given mention into one of two categories: being associated with the main patient (and subject to risk quantification) or not (and assigned risk value NONE). The whole corpus contains 109,386 entities with the risk assigned manually, which depending on whether the Risk value is NONE or not, could serve as negative and positive cases. This, being a binary classification task, was approached through logistic regression.

Firstly, for each mention a feature representation was generated to provide necessary context for the risk classification. This feature set was the same as the risk quantification (see the main body of the article for details). Secondly, a logistic regression model was built using the *glmnet* package [3] in *R*. In order to avoid the detrimental effect of the vast number of features, LASSO regularisation was applied (α set to 1) with λ selected through cross-validation. Based on the

[1] https://taku910.github.io/crfpp/

experiments with the training set, the threshold for the probability score was set at 0.8 and all mentions assigned less than this were labelled as NONE.

2.2.1 Risk quantification

In the last step of the process, all mentions that were not labelled as NONE are subject to risk quantification. Specifically, a CALM model is applied to compute risk score, which is then converted into the LOW, MEDIUM and HIGH categories using predefined thresholds. This task is the topic of the main body of the article.

3 Evaluation

In order to evaluate the end-to-end solution, the models used for the three steps (prepared based on the training data) were run consecutively on the test data documents. Specifically, risk factors recognised at stage 1 were scored at stage 2 and either assigned NONE or quantified at stage 3. Since every stage can introduce its own errors, it is necessary to evaluate the complete workflow to assess the performance in a practical setting.

To this effect the manually-annotated mentions (*true*) were compared to those recognised by the system (*predicted*) on test documents. Two entities were considered to *match* if their spans overlap (i.e. relaxed matching). Based on this, the following measures were computed:

- Overall recall – how many of the true mentions were matched by the predictions,
- Overall precision – how many of the predicted mentions were matching the true ones (ignoring the category and risk value),
- Category accuracy – how many of the matching predicted mentions have the correct category,
- Risk accuracy – how many of the matching predicted mentions have the correct risk value,
- For each of the possible risk levels (NONE/LOW/MEDIUM/HIGH), how many of the true mentions with such risk are:
  - correct – matched by a predicted mention with the same risk value,
  - incorrect – matched by a predicted mention, but with a different risk value,
  - missed – not matched by any prediction.

In order to put these values in context, an inter-annotator agreement (IAA) baseline was computed on the double-annotated documents by treating annotations by one expert as true and the other one's as predicted. The values were aggregated over all annotator pairs and both directions.

Measure	Model	IAA
Overall recall	89.54%	93.00%
Overall precision	94.30%	93.00%
Category accuracy	93.87%	92.11%
Risk accuracy	70.48%	75.49%
None correct	68.52%	76.94%
None incorrect	20.48%	15.47%
None missed	11.00%	7.59%
Low correct	66.15%	73.60%
Low incorrect	24.31%	19.47%
Low missed	9.54%	6.93%
Medium correct	65.92%	67.53%
Medium incorrect	22.46%	24.84%
Medium missed	11.62%	7.63%
High correct	49.79%	67.42%
High incorrect	41.29%	27.39%
High missed	8.92%	5.20%

Table 1: Evaluation results of the end-to-end solution, including the inter-annotator agreement baseline.

Table 3 includes the results. It shows that in terms of overall mention recognition, the system's high precision is not matched by equally satisfactory recall. This obviously affects the later stages of workflow, since when a mention is missed at the NER stage, it cannot be assessed for correctness.

Looking at the accuracy measures, the categories are predicted very well, but risk values pose a larger challenge. Although the performance of risk quantification in isolation was on par with human agreement (see the main body of the article), in the end-to-end evaluation there is more room for improvement. There are two reasons for this. Firstly, to compute the accuracy (as opposed to mean squared error), the risk scores had to be converted to Low, Medium or High, which could increase the error if a border-line value is converted to the wrong category. Secondly, during testing the risk assessment uses the automatically recognised mentions, which are noisier than the true mentions on which the model was trained.

The results for the accuracy at each risk value confirm the observations made in the main body of the article, with the High-ranked risk factors being the hardest to assess properly. This is demonstrated both by a high ratio of factors with *incorrect* value, but also by a significant number of them being missed entirely. Based on this evaluation, improving the mention recall seems to be an obvious direction of development.

Entity normalisation is one of the elements of the end-to-end solution that play a part in the problems discussed above. Firstly, the risk factor recognition relies heavily on features generated from *MetaMap* output, so when it fails to associate a word with an ontology concept, then unless there are other contex-

tual clues, the mention can be missed. Secondly, since the concept IDs define the multi-task structure, the normalisation accuracy is crucial for the risk quantification performance. Unfortunately, this problem is challenging in the case of EHRs due to issues, such as acronyms, loose syntax, ad-hoc abbreviations and misspellings. Replacing the *UMLS MetaMap* with a more tailored solution, optimised for processing clinical records [12] or acronym resolution [8], seems to be a promising direction.

References

[1] Lance De Vine, Mahnoosh Kholghi, Guido Zuccon, Laurianne Sitbon, and Anthony Nguyen. Analysis of Word Embeddings and Sequence Features for Clinical Information Extraction. In *Proceedings of the Australasian Language Technology Association Workshop 2015*, pages 21–30, 2015.

[2] Dina Demner-Fushman, Willie J Rogers, and Alan R Aronson. MetaMap Lite: an evaluation of a new Java implementation of MetaMap. *Journal of the American Medical Informatics Association*, 42(5):841–844, jan 2017.

[3] Jerome Friedman, Trevor Hastie, and Robert Tibshirani. Regularization Paths for Generalized Linear Models via Coordinate Descent. *Journal of Statistical Software*, 33(1), 2010.

[4] A. Hartigan and M. A. Wong. A K-Means Clustering Algorithm. *Journal of the Royal Statistical Society*, 1979.

[5] David M. Jessop, Sam E. Adams, Egon L. Willighagen, Lezan Hawizy, and Peter Murray-Rust. OSCAR4: A flexible architecture for chemical textmining. *Journal of Cheminformatics*, 3(10), 2011.

[6] John Lafferty, Andrew McCallum, and Fernando Pereira. Conditional random fields: Probabilistic models for segmenting and labeling sequence data. *Proceedings of the 18th International Conference on Machine Learning (ICML)*, 2001.

[7] Tomas Mikolov, Kai Chen, Greg Corrado, and Jeffrey Dean. Distributed Representations of Words and Phrases and their Compositionality. In *Advances in Neural Information Processing Systems 26 (NIPS 2013)*, 2013.

[8] N. Okazaki and S. Ananiadou. Building an abbreviation dictionary using a term recognition approach. *Bioinformatics*, 22(24):3089–3095, 2006.

[9] R Core Team. R: A Language and Environment for Statistical Computing, 2013.

[10] Rune Sætre, Kazuhiro Yoshida, Akane Yakushiji, Yusuke Miyao, Yuichiro Matsubayashi, and Tomoko Ohta. AKANE System : Protein-Protein Interaction Pairs in the BioCreAtIvE2 Challenge , PPI-IPS subtask. *Proceedings of the Second BioCreative Challenge Evaluation Workshop*, 2007.

[11] Anja Struyf, Mia Hubert, and Peter Rousseeuw. Clustering in an Object-Oriented Environment. *Journal of Statistical Software*, 1996.

[12] P Thompson and S Ananiadou. HYPHEN: A flexible, hybrid method to map phenotype concept mentions to terminological resources. *Terminology*, 24(1):91–121, 2018.

[13] Yoshimasa Tsuruoka and Jun'ichi Tsujii. Bidirectional inference with the easiest-first strategy for tagging sequence data. *Proceedings of HLT/EMNLP 2005*, 2005.
